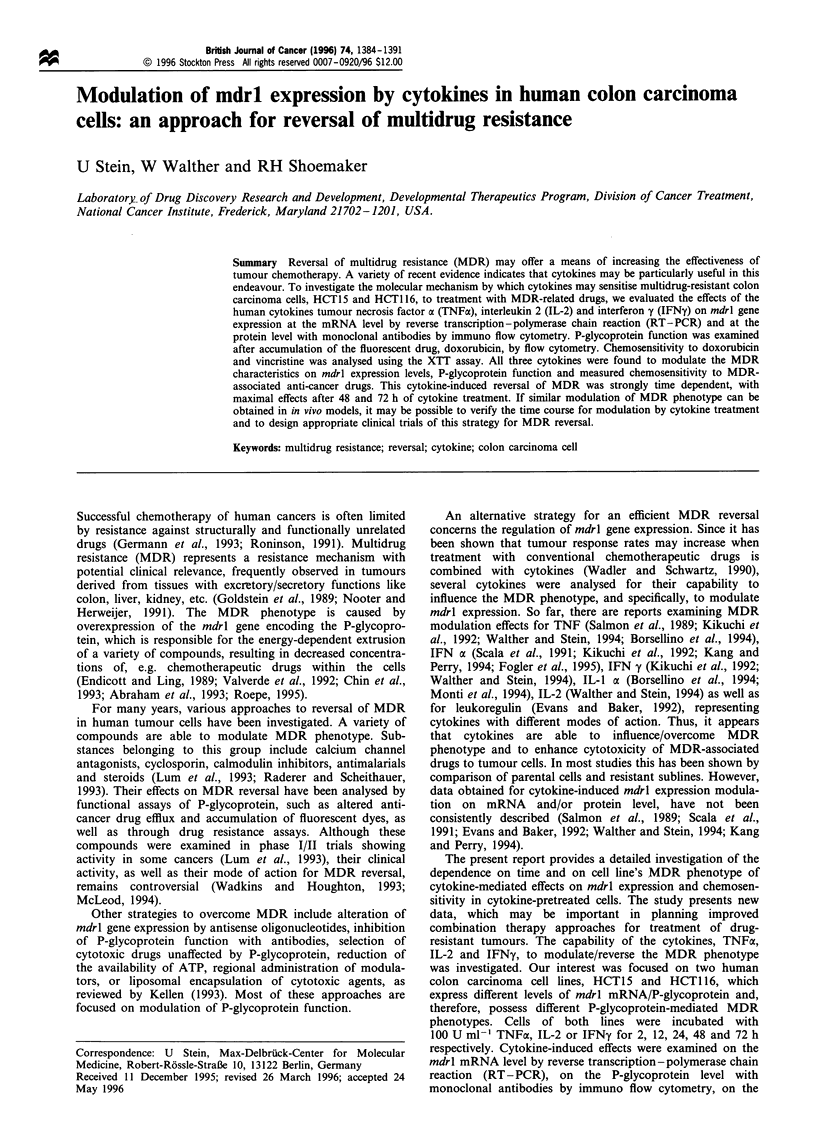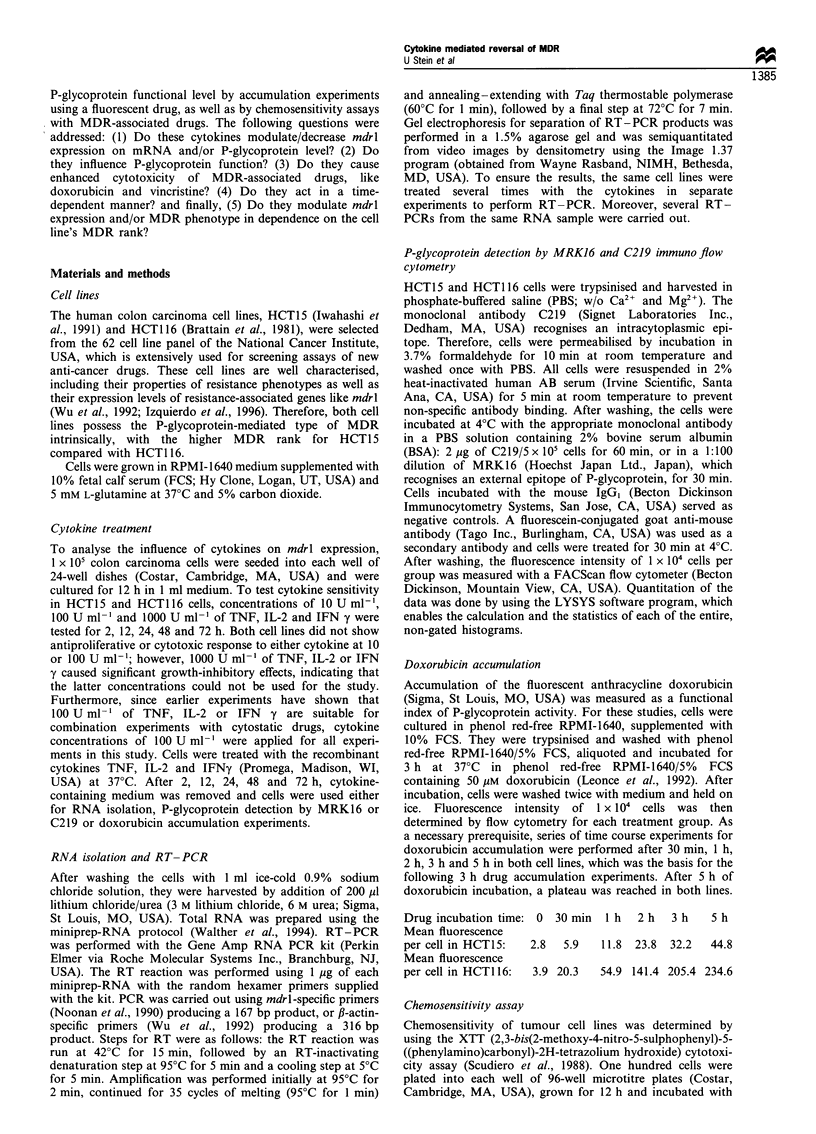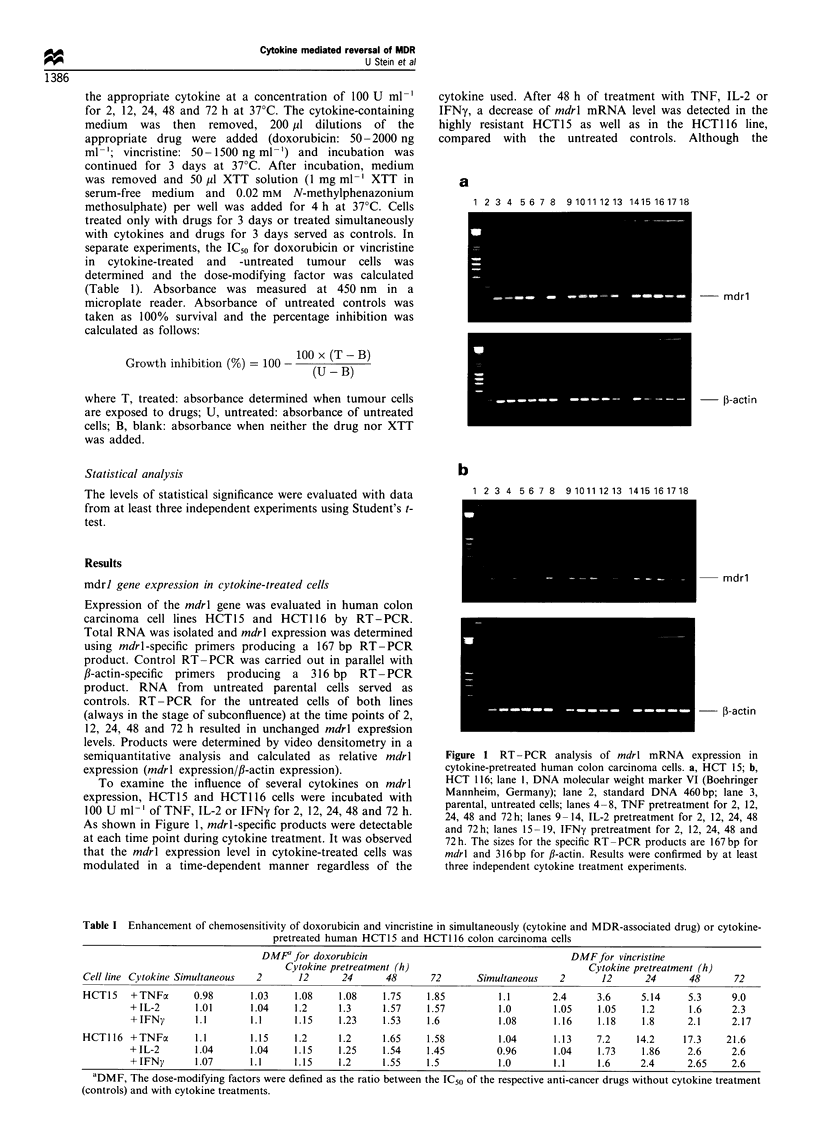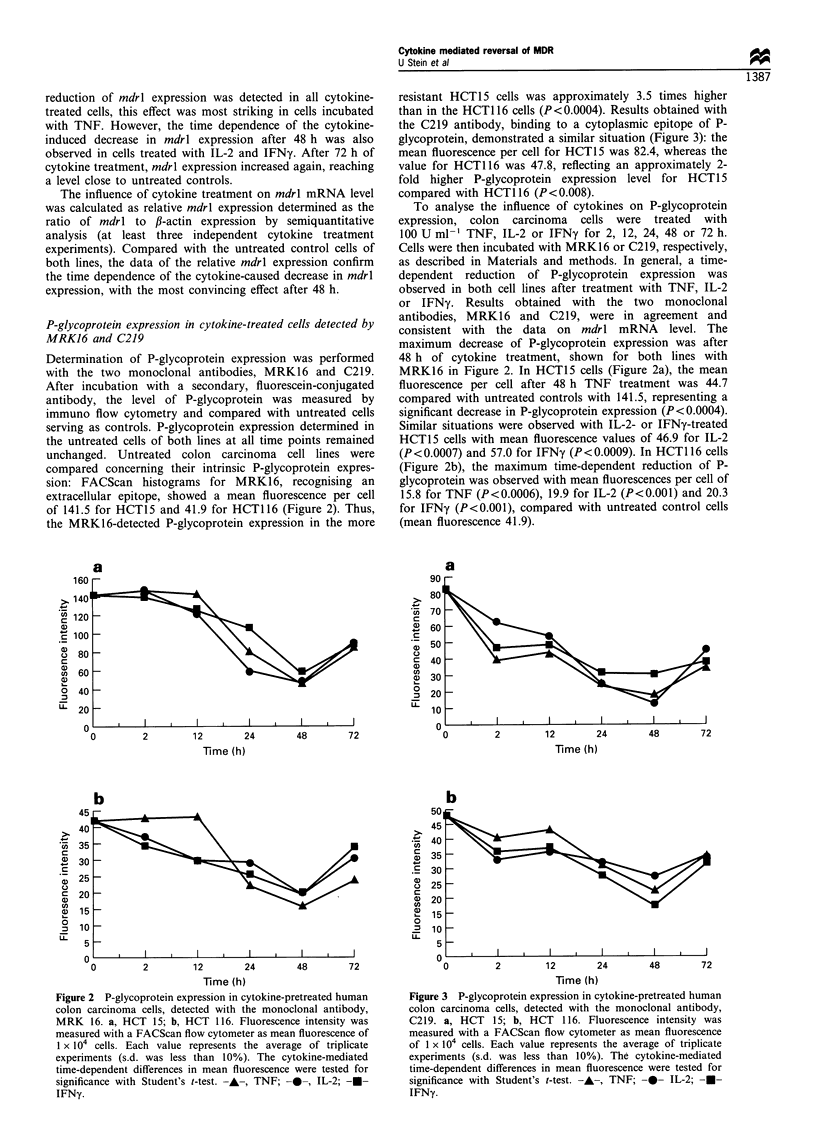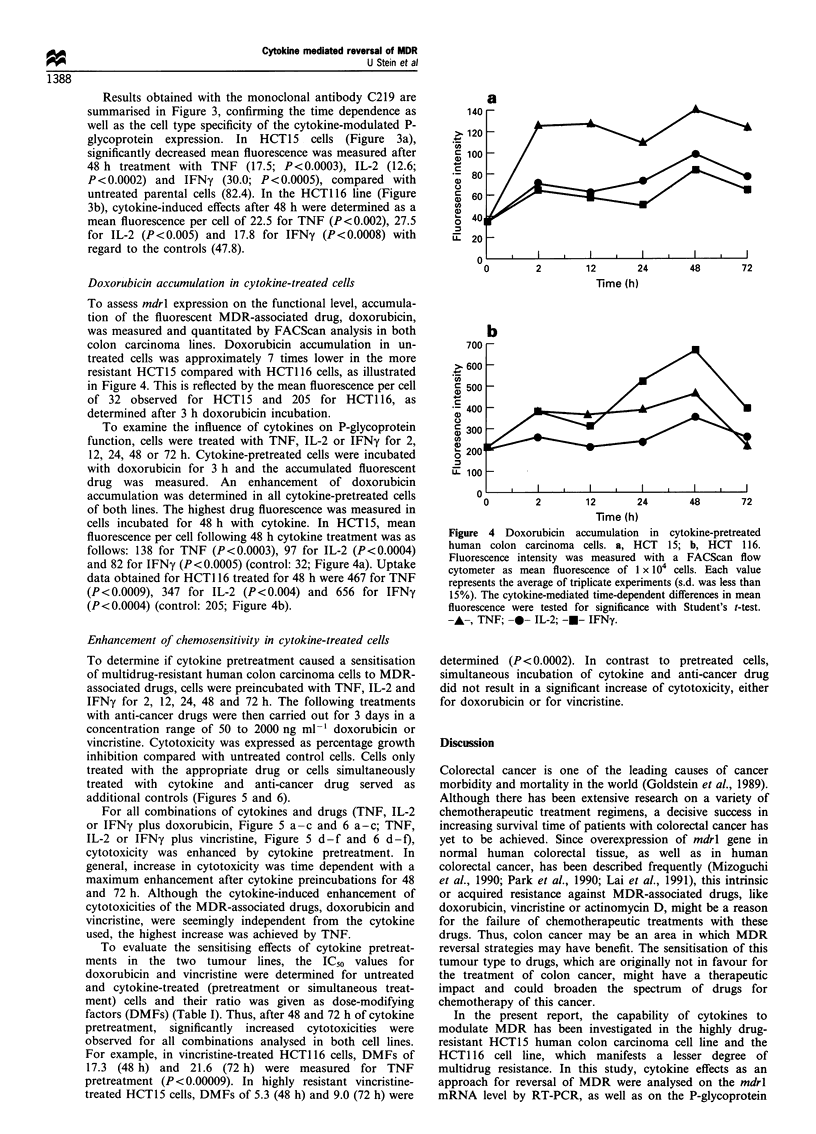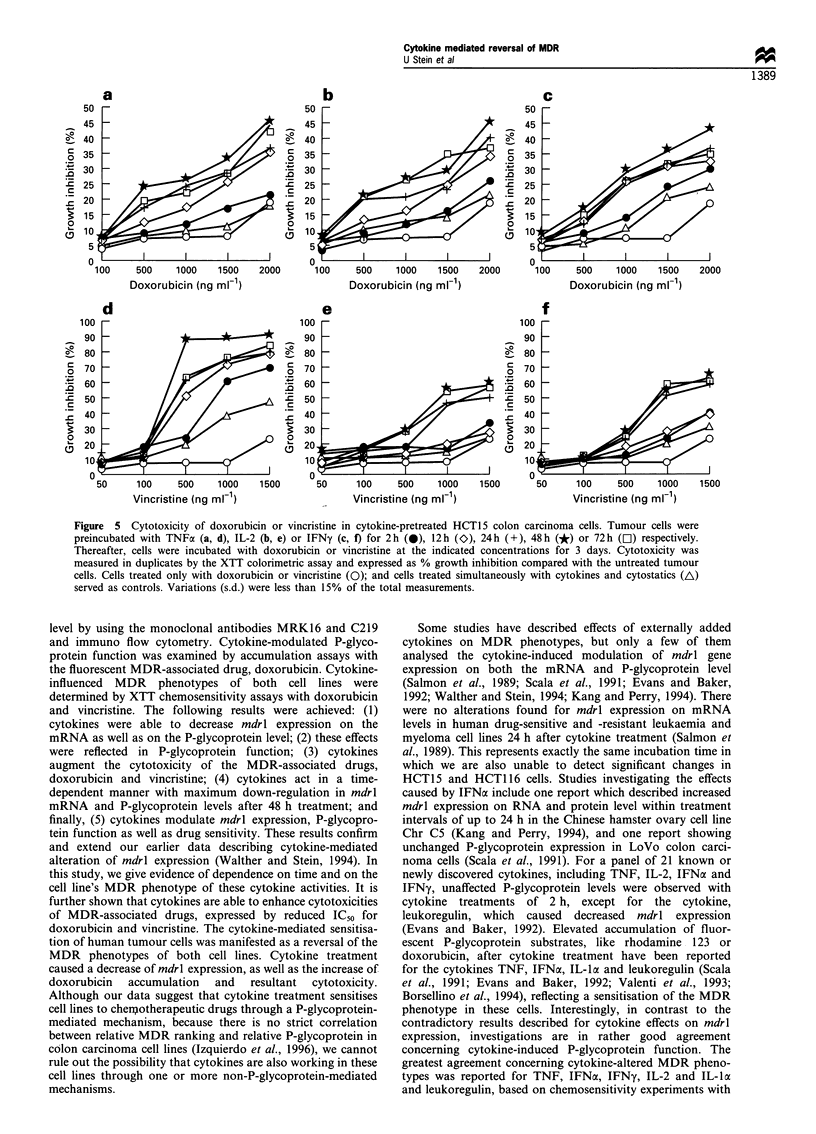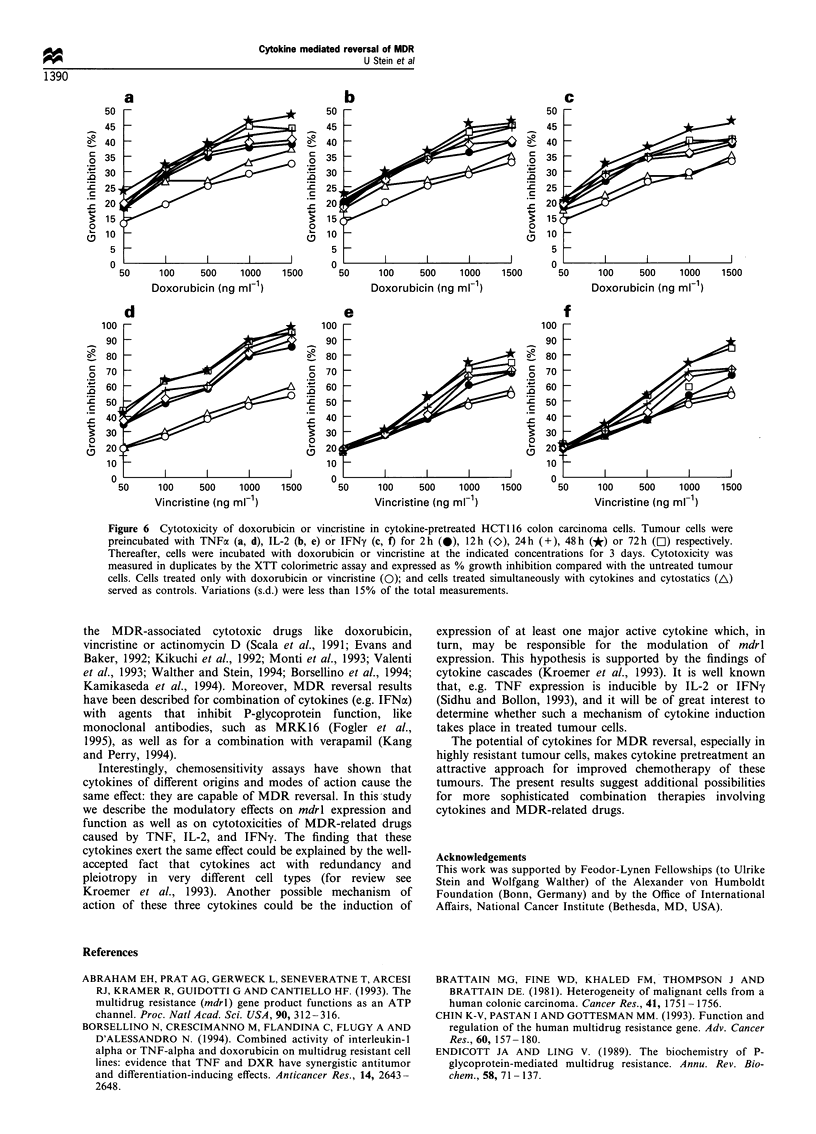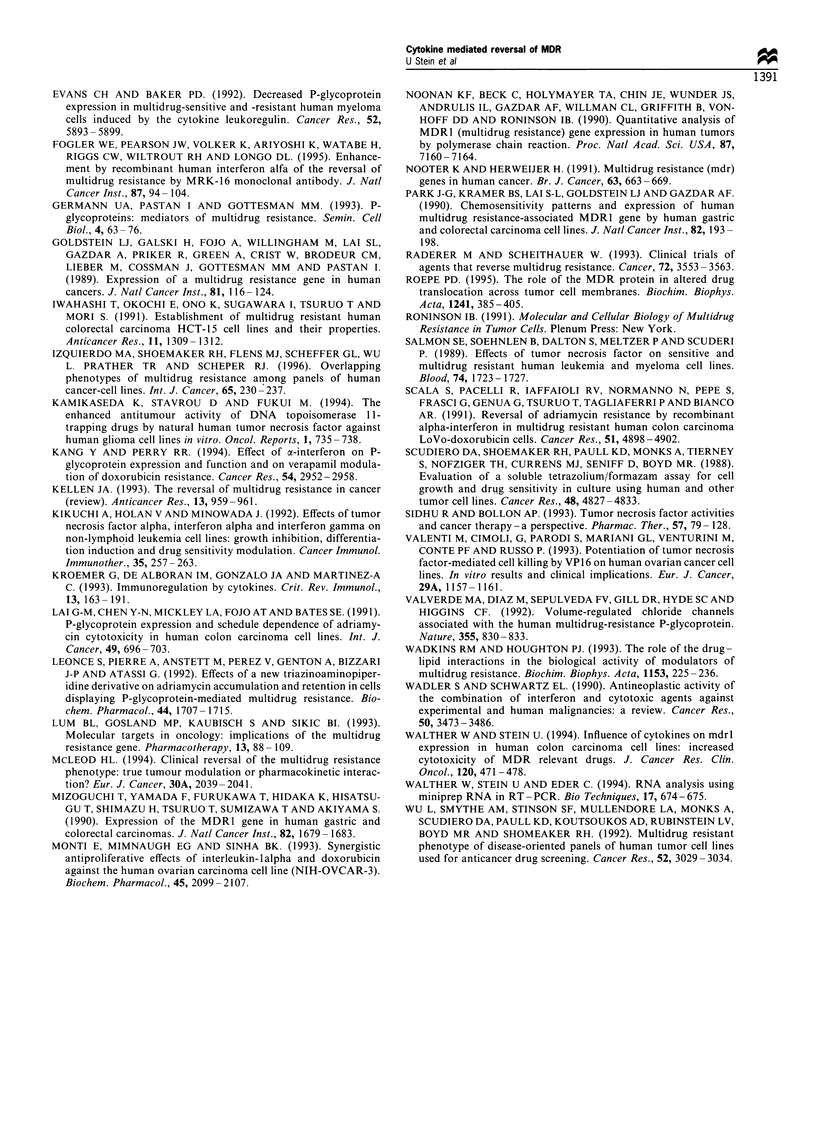# Modulation of mdr1 expression by cytokines in human colon carcinoma cells: an approach for reversal of multidrug resistance.

**DOI:** 10.1038/bjc.1996.553

**Published:** 1996-11

**Authors:** U. Stein, W. Walther, R. H. Shoemaker

**Affiliations:** Laboratory of Drug Discovery Research and Development, Division of Cancer Treatment, National Cancer Institute, Frederick, Maryland 21702-1201, USA.

## Abstract

**Images:**


British Journal of Cancer (1996) 74, 1384-1391
© 1996 Stockton Press All rights reserved 0007-0920/96 $12.00

### Modulation of mdr1 expression by cytokines in human colon carcinoma cells: an approach for reversal of multidrug resistance

U Stein, W Walther and RH Shoemaker

*Laboratory of Drug Discovery Research and Development, Developmental Therapeutics Program, Division of Cancer Treatment, National Cancer Institute, Frederick, Maryland 21702-1201, USA.*

**Summary** Reversal of multidrug resistance (MDR) may offer a means of increasing the effectiveness of tumour chemotherapy. A variety of recent evidence indicates that cytokines may be particularly useful in this endeavour. To investigate the molecular mechanism by which cytokines may sensitise multidrug-resistant colon carcinoma cells, HCT15 and HCT116, to treatment with MDR-related drugs, we evaluated the effects of the human cytokines tumour necrosis factor α (TNFα), interleukin 2 (IL-2) and interferon γ (IFNγ) on *mdr*1 gene expression at the mRNA level by reverse transcription-polymerase chain reaction (RT-PCR) and at the protein level with monoclonal antibodies by immuno flow cytometry. P-glycoprotein function was examined after accumulation of the fluorescent drug, doxorubicin, by flow cytometry. Chemosensitivity to doxorubicin and vincristine was analysed using the XTT assay. All three cytokines were found to modulate the MDR characteristics on *mdr*1 expression levels, P-glycoprotein function and measured chemosensitivity to MDR-associated anti-cancer drugs. This cytokine-induced reversal of MDR was strongly time dependent, with maximal effects after 48 and 72 h of cytokine treatment. If similar modulation of MDR phenotype can be obtained in *in vivo* models, it may be possible to verify the time course for modulation by cytokine treatment and to design appropriate clinical trials of this strategy for MDR reversal.

**Keywords:** multidrug resistance; reversal; cytokine; colon carcinoma cell

Successful chemotherapy of human cancers is often limited by resistance against structurally and functionally unrelated drugs (Germann *et al.*, 1993; Roninson, 1991). Multidrug resistance (MDR) represents a resistance mechanism with potential clinical relevance, frequently observed in tumours derived from tissues with excretory/secretory functions like colon, liver, kidney, etc. (Goldstein *et al.*, 1989; Nooter and Herweijer, 1991). The MDR phenotype is caused by overexpression of the *mdr*1 gene encoding the P-glycoprotein, which is responsible for the energy-dependent extrusion of a variety of compounds, resulting in decreased concentrations of, e.g. chemotherapeutic drugs within the cells (Endicott and Ling, 1989; Valverde *et al.*, 1992; Chin *et al.*, 1993; Abraham *et al.*, 1993; Roepe, 1995).

For many years, various approaches to reversal of MDR in human tumour cells have been investigated. A variety of compounds are able to modulate MDR phenotype. Substances belonging to this group include calcium channel antagonists, cyclosporin, calmodulin inhibitors, antimalarials and steroids (Lum *et al.*, 1993; Raderer and Scheithauer, 1993). Their effects on MDR reversal have been analysed by functional assays of P-glycoprotein, such as altered anticancer drug efflux and accumulation of fluorescent dyes, as well as through drug resistance assays. Although these compounds were examined in phase I/II trials showing activity in some cancers (Lum *et al.*, 1993), their clinical activity, as well as their mode of action for MDR reversal, remains controversial (Wadkins and Houghton, 1993; McLeod, 1994).

Other strategies to overcome MDR include alteration of *mdr*1 gene expression by antisense oligonucleotides, inhibition of P-glycoprotein function with antibodies, selection of cytotoxic drugs unaffected by P-glycoprotein, reduction of the availability of ATP, regional administration of modulators, or liposomal encapsulation of cytotoxic agents, as reviewed by Kellen (1993). Most of these approaches are focused on modulation of P-glycoprotein function.

Correspondence: U Stein, Max-Delbrück-Center for Molecular Medicine, Robert-Rössle-Straße 10, 13122 Berlin, Germany
Received 11 December 1995; revised 26 March 1996; accepted 24 May 1996

An alternative strategy for an efficient MDR reversal concerns the regulation of *mdr*1 gene expression. Since it has been shown that tumour response rates may increase when treatment with conventional chemotherapeutic drugs is combined with cytokines (Wadler and Schwartz, 1990), several cytokines were analysed for their capability to influence the MDR phenotype, and specifically, to modulate *mdr*1 expression. So far, there are reports examining MDR modulation effects for TNF (Salmon *et al.*, 1989; Kikuchi *et al.*, 1992; Walther and Stein, 1994; Borsellino *et al.*, 1994), IFN α (Scala *et al.*, 1991; Kikuchi *et al.*, 1992; Kang and Perry, 1994; Fogler *et al.*, 1995), IFN γ (Kikuchi *et al.*, 1992; Walther and Stein, 1994), IL-1 α (Borsellino *et al.*, 1994; Monti *et al.*, 1994), IL-2 (Walther and Stein, 1994) as well as for leukoregulin (Evans and Baker, 1992), representing cytokines with different modes of action. Thus, it appears that cytokines are able to influence/overcome MDR phenotype and to enhance cytotoxicity of MDR-associated drugs to tumour cells. In most studies this has been shown by comparison of parental cells and resistant sublines. However, data obtained for cytokine-induced *mdr*1 expression modulation on mRNA and/or protein level, have not been consistently described (Salmon *et al.*, 1989; Scala *et al.*, 1991; Evans and Baker, 1992; Walther and Stein, 1994; Kang and Perry, 1994).

The present report provides a detailed investigation of the dependence on time and on cell line's MDR phenotype of cytokine-mediated effects on *mdr*1 expression and chemosensitivity in cytokine-pretreated cells. The study presents new data, which may be important in planning improved combination therapy approaches for treatment of drug-resistant tumours. The capability of the cytokines, TNFα, IL-2 and IFNγ, to modulate/reverse the MDR phenotype was investigated. Our interest was focused on two human colon carcinoma cell lines, HCT15 and HCT116, which express different levels of *mdr*1 mRNA/P-glycoprotein and, therefore, possess different P-glycoprotein-mediated MDR phenotypes. Cells of both lines were incubated with 100 U $ml^{-1}$ TNFα, IL-2 or IFNγ for 2, 12, 24, 48 and 72 h respectively. Cytokine-induced effects were examined on the *mdr*1 mRNA level by reverse transcription-polymerase chain reaction (RT-PCR), on the P-glycoprotein level with monoclonal antibodies by immuno flow cytometry, on the

P-glycoprotein functional level by accumulation experiments using a fluorescent drug, as well as by chemosensitivity assays with MDR-associated drugs. The following questions were addressed: (1) Do these cytokines modulate/decrease *mdr*1 expression on mRNA and/or P-glycoprotein level? (2) Do they influence P-glycoprotein function? (3) Do they cause enhanced cytotoxicity of MDR-associated drugs, like doxorubicin and vincristine? (4) Do they act in a time-dependent manner? and finally, (5) Do they modulate *mdr*1 expression and/or MDR phenotype in dependence on the cell line's MDR rank?

### Materials and methods

### *Cell lines*

The human colon carcinoma cell lines, HCT15 (Iwahashi *et al.*, 1991) and HCT116 (Brattain *et al.*, 1981), were selected from the 62 cell line panel of the National Cancer Institute, USA, which is extensively used for screening assays of new anti-cancer drugs. These cell lines are well characterised, including their properties of resistance phenotypes as well as their expression levels of resistance-associated genes like *mdr*1 (Wu *et al.*, 1992; Izquierdo *et al.*, 1996). Therefore, both cell lines possess the P-glycoprotein-mediated type of MDR intrinsically, with the higher MDR rank for HCT15 compared with HCT116.

Cells were grown in RPMI-1640 medium supplemented with 10% fetal calf serum (FCS; Hy Clone, Logan, UT, USA) and 5 mM L-glutamine at 37°C and 5% carbon dioxide.

### *Cytokine treatment*

To analyse the influence of cytokines on *mdr*1 expression, $1 \times 10^5$ colon carcinoma cells were seeded into each well of 24-well dishes (Costar, Cambridge, MA, USA) and were cultured for 12 h in 1 ml medium. To test cytokine sensitivity in HCT15 and HCT116 cells, concentrations of 10 U $ml^{-1}$, 100 U $ml^{-1}$ and 1000 U $ml^{-1}$ of TNF, IL-2 and IFN $\gamma$ were tested for 2, 12, 24, 48 and 72 h. Both cell lines did not show antiproliferative or cytotoxic response to either cytokine at 10 or 100 U $ml^{-1}$; however, 1000 U $ml^{-1}$ of TNF, IL-2 or IFN $\gamma$ caused significant growth-inhibitory effects, indicating that the latter concentrations could not be used for the study. Furthermore, since earlier experiments have shown that 100 U $ml^{-1}$ of TNF, IL-2 or IFN $\gamma$ are suitable for combination experiments with cytostatic drugs, cytokine concentrations of 100 U $ml^{-1}$ were applied for all experiments in this study. Cells were treated with the recombinant cytokines TNF, IL-2 and IFN$\gamma$ (Promega, Madison, WI, USA) at 37°C. After 2, 12, 24, 48 and 72 h, cytokine-containing medium was removed and cells were used either for RNA isolation, P-glycoprotein detection by MRK16 or C219 or doxorubicin accumulation experiments.

### *RNA isolation and RT–PCR*

After washing the cells with 1 ml ice-cold 0.9% sodium chloride solution, they were harvested by addition of 200 $\mu$l lithium chloride/urea (3 M lithium chloride, 6 M urea; Sigma, St Louis, MO, USA). Total RNA was prepared using the miniprep-RNA protocol (Walther *et al.*, 1994). RT–PCR was performed with the Gene Amp RNA PCR kit (Perkin Elmer via Roche Molecular Systems Inc., Branchburg, NJ, USA). The RT reaction was performed using 1 $\mu$g of each miniprep-RNA with the random hexamer primers supplied with the kit. PCR was carried out using *mdr*1-specific primers (Noonan *et al.*, 1990) producing a 167 bp product, or $\beta$-actin-specific primers (Wu *et al.*, 1992) producing a 316 bp product. Steps for RT were as follows: the RT reaction was run at 42°C for 15 min, followed by an RT-inactivating denaturation step at 95°C for 5 min and a cooling step at 5°C for 5 min. Amplification was performed initially at 95°C for 2 min, continued for 35 cycles of melting (95°C for 1 min) and annealing–extending with *Taq* thermostable polymerase (60°C for 1 min), followed by a final step at 72°C for 7 min. Gel electrophoresis for separation of RT–PCR products was performed in a 1.5% agarose gel and was semiquantitated from video images by densitometry using the Image 1.37 program (obtained from Wayne Rasband, NIMH, Bethesda, MD, USA). To ensure the results, the same cell lines were treated several times with the cytokines in separate experiments to perform RT–PCR. Moreover, several RT–PCRs from the same RNA sample were carried out.

### *P-glycoprotein detection by MRK16 and C219 immuno flow cytometry*

HCT15 and HCT116 cells were trypsinised and harvested in phosphate-buffered saline (PBS; w/o $Ca^{2+}$ and $Mg^{2+}$). The monoclonal antibody C219 (Signet Laboratories Inc., Dedham, MA, USA) recognises an intracytoplasmic epitope. Therefore, cells were permeabilised by incubation in 3.7% formaldehyde for 10 min at room temperature and washed once with PBS. All cells were resuspended in 2% heat-inactivated human AB serum (Irvine Scientific, Santa Ana, CA, USA) for 5 min at room temperature to prevent non-specific antibody binding. After washing, the cells were incubated at 4°C with the appropriate monoclonal antibody in a PBS solution containing 2% bovine serum albumin (BSA): 2 $\mu$g of C219/$5 \times 10^5$ cells for 60 min, or in a 1:100 dilution of MRK16 (Hoechst Japan Ltd., Japan), which recognises an external epitope of P-glycoprotein, for 30 min. Cells incubated with the mouse $IgG_1$ (Becton Dickinson Immunocytometry Systems, San Jose, CA, USA) served as negative controls. A fluorescein-conjugated goat anti-mouse antibody (Tago Inc., Burlingham, CA, USA) was used as a secondary antibody and cells were treated for 30 min at 4°C. After washing, the fluorescence intensity of $1 \times 10^4$ cells per group was measured with a FACScan flow cytometer (Becton Dickinson, Mountain View, CA, USA). Quantitation of the data was done by using the LYSYS software program, which enables the calculation and the statistics of each of the entire, non-gated histograms.

### *Doxorubicin accumulation*

Accumulation of the fluorescent anthracycline doxorubicin (Sigma, St Louis, MO, USA) was measured as a functional index of P-glycoprotein activity. For these studies, cells were cultured in phenol red-free RPMI-1640, supplemented with 10% FCS. They were trypsinised and washed with phenol red-free RPMI-1640/5% FCS, aliquoted and incubated for 3 h at 37°C in phenol red-free RPMI-1640/5% FCS containing 50 $\mu$M doxorubicin (Leonce *et al.*, 1992). After incubation, cells were washed twice with medium and held on ice. Fluorescence intensity of $1 \times 10^4$ cells was then determined by flow cytometry for each treatment group. As a necessary prerequisite, series of time course experiments for doxorubicin accumulation were performed after 30 min, 1 h, 2 h, 3 h and 5 h in both cell lines, which was the basis for the following 3 h drug accumulation experiments. After 5 h of doxorubicin incubation, a plateau was reached in both lines.

| Drug incubation time: | 0 | 30 min | 1 h | 2 h | 3 h | 5 h |
|---|---|---|---|---|---|---|
| Mean fluorescence per cell in HCT15: | 2.8 | 5.9 | 11.8 | 23.8 | 32.2 | 44.8 |
| Mean fluorescence per cell in HCT116: | 3.9 | 20.3 | 54.9 | 141.4 | 205.4 | 234.6 |

### *Chemosensitivity assay*

Chemosensitivity of tumour cell lines was determined by using the XTT (2,3-*bis*(2-methoxy-4-nitro-5-sulphophenyl)-5-((phenylamino)carbonyl)-2H-tetrazolium hydroxide) cytotoxicity assay (Scudiero *et al.*, 1988). One hundred cells were plated into each well of 96-well microtitre plates (Costar, Cambridge, MA, USA), grown for 12 h and incubated with the appropriate cytokine at a concentration of 100 U ml$^{-1}$ for 2, 12, 24, 48 and 72 h at 37°C. The cytokine-containing medium was then removed, 200 $\mu$l dilutions of the appropriate drug were added (doxorubicin: 50–2000 ng ml$^{-1}$; vincristine: 50–1500 ng ml$^{-1}$) and incubation was continued for 3 days at 37°C. After incubation, medium was removed and 50 $\mu$l XTT solution (1 mg ml$^{-1}$ XTT in serum-free medium and 0.02 mM *N*-methylphenazonium methosulphate) per well was added for 4 h at 37°C. Cells treated only with drugs for 3 days or treated simultaneously with cytokines and drugs for 3 days served as controls. In separate experiments, the $IC_{50}$ for doxorubicin or vincristine in cytokine-treated and -untreated tumour cells was determined and the dose-modifying factor was calculated (Table 1). Absorbance was measured at 450 nm in a microplate reader. Absorbance of untreated controls was taken as 100% survival and the percentage inhibition was calculated as follows:

$$\text{Growth inhibition (\%)} = 100 - \frac{100 \times (T - B)}{(U - B)}$$

where T, treated: absorbance determined when tumour cells are exposed to drugs; U, untreated: absorbance of untreated cells; B, blank: absorbance when neither the drug nor XTT was added.

### *Statistical analysis*

The levels of statistical significance were evaluated with data from at least three independent experiments using Student's *t*-test.

### Results

### mdr*1 gene expression in cytokine-treated cells*

Expression of the *mdr*1 gene was evaluated in human colon carcinoma cell lines HCT15 and HCT116 by RT–PCR. Total RNA was isolated and *mdr*1 expression was determined using *mdr*1-specific primers producing a 167 bp RT–PCR product. Control RT–PCR was carried out in parallel with $\beta$-actin-specific primers producing a 316 bp RT–PCR product. RNA from untreated parental cells served as controls. RT–PCR for the untreated cells of both lines (always in the stage of subconfluence) at the time points of 2, 12, 24, 48 and 72 h resulted in unchanged *mdr*1 expression levels. Products were determined by video densitometry in a semiquantitative analysis and calculated as relative *mdr*1 expression (*mdr*1 expression/$\beta$-actin expression).

To examine the influence of several cytokines on *mdr*1 expression, HCT15 and HCT116 cells were incubated with 100 U ml$^{-1}$ of TNF, IL-2 or IFN$\gamma$ for 2, 12, 24, 48 and 72 h. As shown in Figure 1, *mdr*1-specific products were detectable at each time point during cytokine treatment. It was observed that the *mdr*1 expression level in cytokine-treated cells was modulated in a time-dependent manner regardless of the cytokine used. After 48 h of treatment with TNF, IL-2 or IFN$\gamma$, a decrease of *mdr*1 mRNA level was detected in the highly resistant HCT15 as well as in the HCT116 line, compared with the untreated controls. Although the

**Figure 1** RT–PCR analysis of *mdr*1 mRNA expression in cytokine-pretreated human colon carcinoma cells. **a**, HCT 15; **b**, HCT 116; lane 1, DNA molecular weight marker VI (Boehringer Mannheim, Germany); lane 2, standard DNA 460 bp; lane 3, parental, untreated cells; lanes 4–8, TNF pretreatment for 2, 12, 24, 48 and 72 h; lanes 9–14, IL-2 pretreatment for 2, 12, 24, 48 and 72 h; lanes 15–19, IFN$\gamma$ pretreatment for 2, 12, 24, 48 and 72 h. The sizes for the specific RT–PCR products are 167 bp for *mdr*1 and 316 bp for $\beta$-actin. Results were confirmed by at least three independent cytokine treatment experiments.

**Table I** Enhancement of chemosensitivity of doxorubicin and vincristine in simultaneously (cytokine and MDR-associated drug) or cytokine-pretreated human HCT15 and HCT116 colon carcinoma cells

| | | *DMF[a] for doxorubicin* | | | | | | *DMF for vincristine* | | | | | |
|---|---|---|---|---|---|---|---|---|---|---|---|---|---|
| | | | *Cytokine pretreatment (h)* | | | | | | *Cytokine pretreatment (h)* | | | | |
| *Cell line* | *Cytokine* | *Simultaneous* | *2* | *12* | *24* | *48* | *72* | *Simultaneous* | *2* | *12* | *24* | *48* | *72* |
| HCT15 | +TNF$\alpha$ | 0.98 | 1.03 | 1.08 | 1.08 | 1.75 | 1.85 | 1.1 | 2.4 | 3.6 | 5.14 | 5.3 | 9.0 |
| | +IL-2 | 1.01 | 1.04 | 1.2 | 1.3 | 1.57 | 1.57 | 1.0 | 1.05 | 1.05 | 1.2 | 1.6 | 2.3 |
| | +IFN$\gamma$ | 1.1 | 1.1 | 1.15 | 1.23 | 1.53 | 1.6 | 1.08 | 1.16 | 1.18 | 1.8 | 2.1 | 2.17 |
| HCT116 | +TNF$\alpha$ | 1.1 | 1.15 | 1.2 | 1.2 | 1.65 | 1.58 | 1.04 | 1.13 | 7.2 | 14.2 | 17.3 | 21.6 |
| | +IL-2 | 1.04 | 1.04 | 1.15 | 1.25 | 1.54 | 1.45 | 0.96 | 1.04 | 1.73 | 1.86 | 2.6 | 2.6 |
| | +IFN$\gamma$ | 1.07 | 1.1 | 1.15 | 1.2 | 1.55 | 1.5 | 1.0 | 1.1 | 1.6 | 2.4 | 2.65 | 2.6 |

[a]DMF, The dose-modifying factors were defined as the ratio between the $IC_{50}$ of the respective anti-cancer drugs without cytokine treatment (controls) and with cytokine treatments.

reduction of *mdr*1 expression was detected in all cytokine-treated cells, this effect was most striking in cells incubated with TNF. However, the time dependence of the cytokine-induced decrease in *mdr*1 expression after 48 h was also observed in cells treated with IL-2 and IFNγ. After 72 h of cytokine treatment, *mdr*1 expression increased again, reaching a level close to untreated controls.

The influence of cytokine treatment on *mdr*1 mRNA level was calculated as relative *mdr*1 expression determined as the ratio of *mdr*1 to *β*-actin expression by semiquantitative analysis (at least three independent cytokine treatment experiments). Compared with the untreated control cells of both lines, the data of the relative *mdr*1 expression confirm the time dependence of the cytokine-caused decrease in *mdr*1 expression, with the most convincing effect after 48 h.

### *P-glycoprotein expression in cytokine-treated cells detected by MRK16 and C219*

Determination of P-glycoprotein expression was performed with the two monoclonal antibodies, MRK16 and C219. After incubation with a secondary, fluorescein-conjugated antibody, the level of P-glycoprotein was measured by immuno flow cytometry and compared with untreated cells serving as controls. P-glycoprotein expression determined in the untreated cells of both lines at all time points remained unchanged. Untreated colon carcinoma cell lines were compared concerning their intrinsic P-glycoprotein expression: FACScan histograms for MRK16, recognising an extracellular epitope, showed a mean fluorescence per cell of 141.5 for HCT15 and 41.9 for HCT116 (Figure 2). Thus, the MRK16-detected P-glycoprotein expression in the more resistant HCT15 cells was approximately 3.5 times higher than in the HCT116 cells ($P<0.0004$). Results obtained with the C219 antibody, binding to a cytoplasmic epitope of P-glycoprotein, demonstrated a similar situation (Figure 3): the mean fluorescence per cell for HCT15 was 82.4, whereas the value for HCT116 was 47.8, reflecting an approximately 2-fold higher P-glycoprotein expression level for HCT15 compared with HCT116 ($P<0.008$).

To analyse the influence of cytokines on P-glycoprotein expression, colon carcinoma cells were treated with 100 U $ml^{-1}$ TNF, IL-2 or IFNγ for 2, 12, 24, 48 or 72 h. Cells were then incubated with MRK16 or C219, respectively, as described in Materials and methods. In general, a time-dependent reduction of P-glycoprotein expression was observed in both cell lines after treatment with TNF, IL-2 or IFNγ. Results obtained with the two monoclonal antibodies, MRK16 and C219, were in agreement and consistent with the data on *mdr*1 mRNA level. The maximum decrease of P-glycoprotein expression was after 48 h of cytokine treatment, shown for both lines with MRK16 in Figure 2. In HCT15 cells (Figure 2a), the mean fluorescence per cell after 48 h TNF treatment was 44.7 compared with untreated controls with 141.5, representing a significant decrease in P-glycoprotein expression ($P<0.0004$). Similar situations were observed with IL-2- or IFNγ-treated HCT15 cells with mean fluorescence values of 46.9 for IL-2 ($P<0.0007$) and 57.0 for IFNγ ($P<0.0009$). In HCT116 cells (Figure 2b), the maximum time-dependent reduction of P-glycoprotein was observed with mean fluorescences per cell of 15.8 for TNF ($P<0.0006$), 19.9 for IL-2 ($P<0.001$) and 20.3 for IFNγ ($P<0.001$), compared with untreated control cells (mean fluorescence 41.9).

**Figure 2** P-glycoprotein expression in cytokine-pretreated human colon carcinoma cells, detected with the monoclonal antibody, MRK 16. **a**, HCT 15; **b**, HCT 116. Fluorescence intensity was measured with a FACScan flow cytometer as mean fluorescence of $1 \times 10^4$ cells. Each value represents the average of triplicate experiments (s.d. was less than 10%). The cytokine-mediated time-dependent differences in mean fluorescence were tested for significance with Student's *t*-test. –▲–, TNF; –●–, IL-2; –■– IFNγ.

**Figure 3** P-glycoprotein expression in cytokine-pretreated human colon carcinoma cells, detected with the monoclonal antibody, C219. **a**, HCT 15; **b**, HCT 116. Fluorescence intensity was measured with a FACScan flow cytometer as mean fluorescence of $1 \times 10^4$ cells. Each value represents the average of triplicate experiments (s.d. was less than 10%). The cytokine-mediated time-dependent differences in mean fluorescence were tested for significance with Student's *t*-test. –▲–, TNF; –●– IL-2; –■– IFNγ.

Results obtained with the monoclonal antibody C219 are summarised in Figure 3, confirming the time dependence as well as the cell type specificity of the cytokine-modulated P-glycoprotein expression. In HCT15 cells (Figure 3a), significantly decreased mean fluorescence was measured after 48 h treatment with TNF (17.5; $P<0.0003$), IL-2 (12.6; $P<0.0002$) and IFNγ (30.0; $P<0.0005$), compared with untreated parental cells (82.4). In the HCT116 line (Figure 3b), cytokine-induced effects after 48 h were determined as a mean fluorescence per cell of 22.5 for TNF ($P<0.002$), 27.5 for IL-2 ($P<0.005$) and 17.8 for IFNγ ($P<0.0008$) with regard to the controls (47.8).

*Doxorubicin accumulation in cytokine-treated cells*

To assess *mdr*1 expression on the functional level, accumulation of the fluorescent MDR-associated drug, doxorubicin, was measured and quantitated by FACScan analysis in both colon carcinoma lines. Doxorubicin accumulation in untreated cells was approximately 7 times lower in the more resistant HCT15 compared with HCT116 cells, as illustrated in Figure 4. This is reflected by the mean fluorescence per cell of 32 observed for HCT15 and 205 for HCT116, as determined after 3 h doxorubicin incubation.

To examine the influence of cytokines on P-glycoprotein function, cells were treated with TNF, IL-2 or IFNγ for 2, 12, 24, 48 or 72 h. Cytokine-pretreated cells were incubated with doxorubicin for 3 h and the accumulated fluorescent drug was measured. An enhancement of doxorubicin accumulation was determined in all cytokine-pretreated cells of both lines. The highest drug fluorescence was measured in cells incubated for 48 h with cytokine. In HCT15, mean fluorescence per cell following 48 h cytokine treatment was as follows: 138 for TNF ($P<0.0003$), 97 for IL-2 ($P<0.0004$) and 82 for IFNγ ($P<0.0005$) (control: 32; Figure 4a). Uptake data obtained for HCT116 treated for 48 h were 467 for TNF ($P<0.0009$), 347 for IL-2 ($P<0.004$) and 656 for IFNγ ($P<0.0004$) (control: 205; Figure 4b).

**Figure 4** Doxorubicin accumulation in cytokine-pretreated human colon carcinoma cells. **a**, HCT 15; **b**, HCT 116. Fluorescence intensity was measured with a FACScan flow cytometer as mean fluorescence of $1 \times 10^4$ cells. Each value represents the average of triplicate experiments (s.d. was less than 15%). The cytokine-mediated time-dependent differences in mean fluorescence were tested for significance with Student's *t*-test. -▲-, TNF; -●- IL-2; -■- IFNγ.

*Enhancement of chemosensitivity in cytokine-treated cells*

To determine if cytokine pretreatment caused a sensitisation of multidrug-resistant human colon carcinoma cells to MDR-associated drugs, cells were preincubated with TNF, IL-2 and IFNγ for 2, 12, 24, 48 and 72 h. The following treatments with anti-cancer drugs were then carried out for 3 days in a concentration range of 50 to 2000 ng $ml^{-1}$ doxorubicin or vincristine. Cytotoxicity was expressed as percentage growth inhibition compared with untreated control cells. Cells only treated with the appropriate drug or cells simultaneously treated with cytokine and anti-cancer drug served as additional controls (Figures 5 and 6).

For all combinations of cytokines and drugs (TNF, IL-2 or IFNγ plus doxorubicin, Figure 5 a–c and 6 a–c; TNF, IL-2 or IFNγ plus vincristine, Figure 5 d–f and 6 d–f), cytotoxicity was enhanced by cytokine pretreatment. In general, increase in cytotoxicity was time dependent with a maximum enhancement after cytokine preincubations for 48 and 72 h. Although the cytokine-induced enhancement of cytotoxicities of the MDR-associated drugs, doxorubicin and vincristine, were seemingly independent from the cytokine used, the highest increase was achieved by TNF.

To evaluate the sensitising effects of cytokine pretreatments in the two tumour lines, the $IC_{50}$ values for doxorubicin and vincristine were determined for untreated and cytokine-treated (pretreatment or simultaneous treatment) cells and their ratio was given as dose-modifying factors (DMFs) (Table I). Thus, after 48 and 72 h of cytokine pretreatment, significantly increased cytotoxicities were observed for all combinations analysed in both cell lines. For example, in vincristine-treated HCT116 cells, DMFs of 17.3 (48 h) and 21.6 (72 h) were measured for TNF pretreatment ($P<0.00009$). In highly resistant vincristine-treated HCT15 cells, DMFs of 5.3 (48 h) and 9.0 (72 h) were determined ($P<0.0002$). In contrast to pretreated cells, simultaneous incubation of cytokine and anti-cancer drug did not result in a significant increase of cytotoxicity, either for doxorubicin or for vincristine.

### Discussion

Colorectal cancer is one of the leading causes of cancer morbidity and mortality in the world (Goldstein *et al.*, 1989). Although there has been extensive research on a variety of chemotherapeutic treatment regimens, a decisive success in increasing survival time of patients with colorectal cancer has yet to be achieved. Since overexpression of *mdr*1 gene in normal human colorectal tissue, as well as in human colorectal cancer, has been described frequently (Mizoguchi *et al.*, 1990; Park *et al.*, 1990; Lai *et al.*, 1991), this intrinsic or acquired resistance against MDR-associated drugs, like doxorubicin, vincristine or actinomycin D, might be a reason for the failure of chemotherapeutic treatments with these drugs. Thus, colon cancer may be an area in which MDR reversal strategies may have benefit. The sensitisation of this tumour type to drugs, which are originally not in favour for the treatment of colon cancer, might have a therapeutic impact and could broaden the spectrum of drugs for chemotherapy of this cancer.

In the present report, the capability of cytokines to modulate MDR has been investigated in the highly drug-resistant HCT15 human colon carcinoma cell line and the HCT116 cell line, which manifests a lesser degree of multidrug resistance. In this study, cytokine effects as an approach for reversal of MDR were analysed on the *mdr*1 mRNA level by RT-PCR, as well as on the P-glycoprotein

**Figure 5** Cytotoxicity of doxorubicin or vincristine in cytokine-pretreated HCT15 colon carcinoma cells. Tumour cells were preincubated with TNFα (**a**, **d**), IL-2 (**b**, **e**) or IFNγ (**c**, **f**) for 2 h (●), 12 h (◇), 24 h (+), 48 h (★) or 72 h (□) respectively. Thereafter, cells were incubated with doxorubicin or vincristine at the indicated concentrations for 3 days. Cytotoxicity was measured in duplicates by the XTT colorimetric assay and expressed as % growth inhibition compared with the untreated tumour cells. Cells treated only with doxorubicin or vincristine (○); and cells treated simultaneously with cytokines and cytostatics (△) served as controls. Variations (s.d.) were less than 15% of the total measurements.

level by using the monoclonal antibodies MRK16 and C219 and immuno flow cytometry. Cytokine-modulated P-glycoprotein function was examined by accumulation assays with the fluorescent MDR-associated drug, doxorubicin. Cytokine-influenced MDR phenotypes of both cell lines were determined by XTT chemosensitivity assays with doxorubicin and vincristine. The following results were achieved: (1) cytokines were able to decrease *mdr*1 expression on the mRNA as well as on the P-glycoprotein level; (2) these effects were reflected in P-glycoprotein function; (3) cytokines augment the cytotoxicity of the MDR-associated drugs, doxorubicin and vincristine; (4) cytokines act in a time-dependent manner with maximum down-regulation in *mdr*1 mRNA and P-glycoprotein levels after 48 h treatment; and finally, (5) cytokines modulate *mdr*1 expression, P-glycoprotein function as well as drug sensitivity. These results confirm and extend our earlier data describing cytokine-mediated alteration of *mdr*1 expression (Walther and Stein, 1994). In this study, we give evidence of dependence on time and on the cell line's MDR phenotype of these cytokine activities. It is further shown that cytokines are able to enhance cytotoxicities of MDR-associated drugs, expressed by reduced $IC_{50}$ for doxorubicin and vincristine. The cytokine-mediated sensitisation of human tumour cells was manifested as a reversal of the MDR phenotypes of both cell lines. Cytokine treatment caused a decrease of *mdr*1 expression, as well as the increase of doxorubicin accumulation and resultant cytotoxicity. Although our data suggest that cytokine treatment sensitises cell lines to chemotherapeutic drugs through a P-glycoprotein-mediated mechanism, because there is no strict correlation between relative MDR ranking and relative P-glycoprotein in colon carcinoma cell lines (Izquierdo *et al*., 1996), we cannot rule out the possibility that cytokines are also working in these cell lines through one or more non-P-glycoprotein-mediated mechanisms.

Some studies have described effects of externally added cytokines on MDR phenotypes, but only a few of them analysed the cytokine-induced modulation of *mdr*1 gene expression on both the mRNA and P-glycoprotein level (Salmon *et al*., 1989; Scala *et al*., 1991; Evans and Baker, 1992; Walther and Stein, 1994; Kang and Perry, 1994). There were no alterations found for *mdr*1 expression on mRNA levels in human drug-sensitive and -resistant leukaemia and myeloma cell lines 24 h after cytokine treatment (Salmon *et al*., 1989). This represents exactly the same incubation time in which we are also unable to detect significant changes in HCT15 and HCT116 cells. Studies investigating the effects caused by IFNα include one report which described increased *mdr*1 expression on RNA and protein level within treatment intervals of up to 24 h in the Chinese hamster ovary cell line Chr C5 (Kang and Perry, 1994), and one report showing unchanged P-glycoprotein expression in LoVo colon carcinoma cells (Scala *et al*., 1991). For a panel of 21 known or newly discovered cytokines, including TNF, IL-2, IFNα and IFNγ, unaffected P-glycoprotein levels were observed with cytokine treatments of 2 h, except for the cytokine, leukoregulin, which caused decreased *mdr*1 expression (Evans and Baker, 1992). Elevated accumulation of fluorescent P-glycoprotein substrates, like rhodamine 123 or doxorubicin, after cytokine treatment have been reported for the cytokines TNF, IFNα, IL-1α and leukoregulin (Scala *et al*., 1991; Evans and Baker, 1992; Valenti *et al*., 1993; Borsellino *et al*., 1994), reflecting a sensitisation of the MDR phenotype in these cells. Interestingly, in contrast to the contradictory results described for cytokine effects on *mdr*1 expression, investigations are in rather good agreement concerning cytokine-induced P-glycoprotein function. The greatest agreement concerning cytokine-altered MDR phenotypes was reported for TNF, IFNα, IFNγ, IL-2 and IL-1α and leukoregulin, based on chemosensitivity experiments with

**Figure 6** Cytotoxicity of doxorubicin or vincristine in cytokine-pretreated HCT116 colon carcinoma cells. Tumour cells were preincubated with TNFα (**a**, **d**), IL-2 (**b**, **e**) or IFNγ (**c**, **f**) for 2h (●), 12h (◇), 24h (+), 48h (★) or 72h (□) respectively. Thereafter, cells were incubated with doxorubicin or vincristine at the indicated concentrations for 3 days. Cytotoxicity was measured in duplicates by the XTT colorimetric assay and expressed as % growth inhibition compared with the untreated tumour cells. Cells treated only with doxorubicin or vincristine (○); and cells treated simultaneously with cytokines and cytostatics (△) served as controls. Variations (s.d.) were less than 15% of the total measurements.

the MDR-associated cytotoxic drugs like doxorubicin, vincristine or actinomycin D (Scala *et al.*, 1991; Evans and Baker, 1992; Kikuchi *et al.*, 1992; Monti *et al.*, 1993; Valenti *et al.*, 1993; Walther and Stein, 1994; Borsellino *et al.*, 1994; Kamikaseda *et al.*, 1994). Moreover, MDR reversal results have been described for combination of cytokines (e.g. IFNα) with agents that inhibit P-glycoprotein function, like monoclonal antibodies, such as MRK16 (Fogler *et al.*, 1995), as well as for a combination with verapamil (Kang and Perry, 1994).

Interestingly, chemosensitivity assays have shown that cytokines of different origins and modes of action cause the same effect: they are capable of MDR reversal. In this study we describe the modulatory effects on *mdr*1 expression and function as well as on cytotoxicities of MDR-related drugs caused by TNF, IL-2, and IFNγ. The finding that these cytokines exert the same effect could be explained by the well-accepted fact that cytokines act with redundancy and pleiotropy in very different cell types (for review see Kroemer *et al.*, 1993). Another possible mechanism of action of these three cytokines could be the induction of expression of at least one major active cytokine which, in turn, may be responsible for the modulation of *mdr*1 expression. This hypothesis is supported by the findings of cytokine cascades (Kroemer *et al.*, 1993). It is well known that, e.g. TNF expression is inducible by IL-2 or IFNγ (Sidhu and Bollon, 1993), and it will be of great interest to determine whether such a mechanism of cytokine induction takes place in treated tumour cells.

The potential of cytokines for MDR reversal, especially in highly resistant tumour cells, makes cytokine pretreatment an attractive approach for improved chemotherapy of these tumours. The present results suggest additional possibilities for more sophisticated combination therapies involving cytokines and MDR-related drugs.

**Acknowledgements**
This work was supported by Feodor-Lynen Fellowships (to Ulrike Stein and Wolfgang Walther) of the Alexander von Humboldt Foundation (Bonn, Germany) and by the Office of International Affairs, National Cancer Institute (Bethesda, MD, USA).

### References

ABRAHAM EH, PRAT AG, GERWECK L, SENEVERATNE T, ARCESI RJ, KRAMER R, GUIDOTTI G AND CANTIELLO HF. (1993). The multidrug resistance (*mdr*l) gene product functions as an ATP channel. *Proc. Natl Acad. Sci. USA*, **90**, 312–316.

BORSELLINO N, CRESCIMANNO M, FLANDINA C, FLUGY A AND D'ALESSANDRO N. (1994). Combined activity of interleukin-1 alpha or TNF-alpha and doxorubicin on multidrug resistant cell lines: evidence that TNF and DXR have synergistic antitumor and differentiation-inducing effects. *Anticancer Res.*, **14**, 2643–2648.

BRATTAIN MG, FINE WD, KHALED FM, THOMPSON J AND BRATTAIN DE. (1981). Heterogeneity of malignant cells from a human colonic carcinoma. *Cancer Res.*, **41**, 1751–1756.

CHIN K-V, PASTAN I AND GOTTESMAN MM. (1993). Function and regulation of the human multidrug resistance gene. *Adv. Cancer Res.*, **60**, 157–180.

ENDICOTT JA AND LING V. (1989). The biochemistry of P-glycoprotein-mediated multidrug resistance. *Annu. Rev. Biochem.*, **58**, 71–137.

EVANS CH AND BAKER PD. (1992). Decreased P-glycoprotein expression in multidrug-sensitive and -resistant human myeloma cells induced by the cytokine leukoregulin. *Cancer Res.*, **52,** 5893-5899.

FOGLER WE, PEARSON JW, VOLKER K, ARIYOSHI K, WATABE H, RIGGS CW, WILTROUT RH AND LONGO DL. (1995). Enhancement by recombinant human interferon alfa of the reversal of multidrug resistance by MRK-16 monoclonal antibody. *J. Natl Cancer Inst.*, **87,** 94-104.

GERMANN UA, PASTAN I AND GOTTESMAN MM. (1993). P-glycoproteins: mediators of multidrug resistance. *Semin. Cell Biol.*, **4,** 63-76.

GOLDSTEIN LJ, GALSKI H, FOJO A, WILLINGHAM M, LAI SL, GAZDAR A, PRIKER R, GREEN A, CRIST W, BRODEUR CM, LIEBER M, COSSMAN J, GOTTESMAN MM AND PASTAN I. (1989). Expression of a multidrug resistance gene in human cancers. *J. Natl Cancer Inst.*, **81,** 116-124.

IWAHASHI T, OKOCHI E, ONO K, SUGAWARA I, TSURUO T AND MORI S. (1991). Establishment of multidrug resistant human colorectal carcinoma HCT-15 cell lines and their properties. *Anticancer Res.*, **11,** 1309-1312.

IZQUIERDO MA, SHOEMAKER RH, FLENS MJ, SCHEFFER GL, WU L. PRATHER TR AND SCHEPER RJ. (1996). Overlapping phenotypes of multidrug resistance among panels of human cancer-cell lines. *Int. J. Cancer*, **65,** 230-237.

KAMIKASEDA K, STAVROU D AND FUKUI M. (1994). The enhanced antitumour activity of DNA topoisomerase 11-trapping drugs by natural human tumor necrosis factor against human glioma cell lines *in vitro*. *Oncol. Reports*, **1,** 735-738.

KANG Y AND PERRY RR. (1994). Effect of α-interferon on P-glycoprotein expression and function and on verapamil modulation of doxorubicin resistance. *Cancer Res.*, **54,** 2952-2958.

KELLEN JA. (1993). The reversal of multidrug resistance in cancer (review). *Anticancer Res.*, **13,** 959-961.

KIKUCHI A, HOLAN V AND MINOWADA J. (1992). Effects of tumor necrosis factor alpha, interferon alpha and interferon gamma on non-lymphoid leukemia cell lines: growth inhibition, differentiation induction and drug sensitivity modulation. *Cancer Immunol. Immunother.*, **35,** 257-263.

KROEMER G, DE ALBORAN IM, GONZALO JA AND MARTINEZ-A C. (1993). Immunoregulation by cytokines. *Crit. Rev. Immunol.*, **13,** 163-191.

LAI G-M, CHEN Y-N, MICKLEY LA, FOJO AT AND BATES SE. (1991). P-glycoprotein expression and schedule dependence of adriamycin cytotoxicity in human colon carcinoma cell lines. *Int. J. Cancer*, **49,** 696-703.

LEONCE S, PIERRE A, ANSTETT M, PEREZ V, GENTON A, BIZZARI J-P AND ATASSI G. (1992). Effects of a new triazinoaminopiperidine derivative on adriamycin accumulation and retention in cells displaying P-glycoprotein-mediated multidrug resistance. *Biochem. Pharmacol.*, **44,** 1707-1715.

LUM BL, GOSLAND MP, KAUBISCH S AND SIKIC BI. (1993). Molecular targets in oncology: implications of the multidrug resistance gene. *Pharmacotherapy*, **13,** 88-109.

McLEOD HL. (1994). Clinical reversal of the multidrug resistance phenotype: true tumour modulation or pharmacokinetic interaction? *Eur. J. Cancer*, **30A,** 2039-2041.

MIZOGUCHI T, YAMADA F, FURUKAWA T, HIDAKA K, HISATSUGU T, SHIMAZU H, TSURUO T, SUMIZAWA T AND AKIYAMA S. (1990). Expression of the MDR1 gene in human gastric and colorectal carcinomas. *J. Natl Cancer Inst.*, **82,** 1679-1683.

MONTI E, MIMNAUGH EG AND SINHA BK. (1993). Synergistic antiproliferative effects of interleukin-1alpha and doxorubicin against the human ovarian carcinoma cell line (NIH-OVCAR-3). *Biochem. Pharmacol.*, **45,** 2099-2107.

NOONAN KF, BECK C, HOLYMAYER TA, CHIN JE, WUNDER JS, ANDRULIS IL, GAZDAR AF, WILLMAN CL, GRIFFITH B, VON-HOFF DD AND RONINSON IB. (1990). Quantitative analysis of MDR1 (multidrug resistance) gene expression in human tumors by polymerase chain reaction. *Proc. Natl Acad. Sci. USA*, **87,** 7160-7164.

NOOTER K AND HERWEIJER H. (1991). Multidrug resistance (mdr) genes in human cancer. *Br. J. Cancer*, **63,** 663-669.

PARK J-G, KRAMER BS, LAI S-L, GOLDSTEIN LJ AND GAZDAR AF. (1990). Chemosensitivity patterns and expression of human multidrug resistance-associated MDR1 gene by human gastric and colorectal carcinoma cell lines. *J. Natl Cancer Inst.*, **82,** 193-198.

RADERER M AND SCHEITHAUER W. (1993). Clinical trials of agents that reverse multidrug resistance. *Cancer*, **72,** 3553-3563.

ROEPE PD. (1995). The role of the MDR protein in altered drug translocation across tumor cell membranes. *Biochim. Biophys. Acta*, **1241,** 385-405.

RONINSON IB. (1991). *Molecular and Cellular Biology of Multidrug Resistance in Tumor Cells.* Plenum Press: New York.

SALMON SE, SOEHNLEN B, DALTON S, MELTZER P AND SCUDERI P. (1989). Effects of tumor necrosis factor on sensitive and multidrug resistant human leukemia and myeloma cell lines. *Blood*, **74,** 1723-1727.

SCALA S, PACELLI R, IAFFAIOLI RV, NORMANNO N, PEPE S, FRASCI G, GENUA G, TSURUO T, TAGLIAFERRI P AND BIANCO AR. (1991). Reversal of adriamycin resistance by recombinant alpha-interferon in multidrug resistant human colon carcinoma LoVo-doxorubicin cells. *Cancer Res.*, **51,** 4898-4902.

SCUDIERO DA, SHOEMAKER RH, PAULL KD, MONKS A, TIERNEY S, NOFZIGER TH, CURRENS MJ, SENIFF D, BOYD MR. (1988). Evaluation of a soluble tetrazolium/formazam assay for cell growth and drug sensitivity in culture using human and other tumor cell lines. *Cancer Res.*, **48,** 4827-4833.

SIDHU R AND BOLLON AP. (1993). Tumor necrosis factor activities and cancer therapy - a perspective. *Pharmac. Ther.*, **57,** 79-128.

VALENTI M, CIMOLI, G, PARODI S, MARIANI GL, VENTURINI M, CONTE PF AND RUSSO P. (1993). Potentiation of tumor necrosis factor-mediated cell killing by VP16 on human ovarian cancer cell lines. *In vitro* results and clinical implications. *Eur. J. Cancer*, **29A,** 1157-1161.

VALVERDE MA, DIAZ M, SEPULVEDA FV, GILL DR, HYDE SC AND HIGGINS CF. (1992). Volume-regulated chloride channels associated with the human multidrug-resistance P-glycoprotein. *Nature*, **355,** 830-833.

WADKINS RM AND HOUGHTON PJ. (1993). The role of the drug-lipid interactions in the biological activity of modulators of multidrug resistance. *Biochim. Biophys. Acta*, **1153,** 225-236.

WADLER S AND SCHWARTZ EL. (1990). Antineoplastic activity of the combination of interferon and cytotoxic agents against experimental and human malignancies: a review. *Cancer Res.*, **50,** 3473-3486.

WALTHER W AND STEIN U. (1994). Influence of cytokines on mdr1 expression in human colon carcinoma cell lines: increased cytotoxicity of MDR relevant drugs. *J. Cancer Res. Clin. Oncol.*, **120,** 471-478.

WALTHER W, STEIN U AND EDER C. (1994). RNA analysis using miniprep RNA in RT-PCR. *Bio Techniques*, **17,** 674-675.

WU L, SMYTHE AM, STINSON SF, MULLENDORE LA, MONKS A, SCUDIERO DA, PAULL KD, KOUTSOUKOS AD, RUBINSTEIN LV, BOYD MR AND SHOMEAKER RH. (1992). Multidrug resistant phenotype of disease-oriented panels of human tumor cell lines used for anticancer drug screening. *Cancer Res.*, **52,** 3029-3034.